# Sex and Death: The Effects of Innate Immune Factors on the Sexual Reproduction of Malaria Parasites

**DOI:** 10.1371/journal.ppat.1001309

**Published:** 2011-03-03

**Authors:** Ricardo S. Ramiro, João Alpedrinha, Lucy Carter, Andy Gardner, Sarah E. Reece

**Affiliations:** 1 Institutes of Evolution, Immunology and Infection Research, University of Edinburgh, Edinburgh, United Kingdom; 2 Department of Zoology, University of Oxford, Oxford, United Kingdom; 3 Instituto Gulbenkian de Ciência, Oeiras, Portugal; 4 Balliol College, University of Oxford, Oxford, United Kingdom; 5 Centre for Immunity, Infection and Evolution, University of Edinburgh, Edinburgh, United Kingdom; Faculdade de Medicina da Universidade de Lisboa, Portugal

## Abstract

Malaria parasites must undergo a round of sexual reproduction in the blood meal of a mosquito vector to be transmitted between hosts. Developing a transmission-blocking intervention to prevent parasites from mating is a major goal of biomedicine, but its effectiveness could be compromised if parasites can compensate by simply adjusting their sex allocation strategies. Recently, the application of evolutionary theory for sex allocation has been supported by experiments demonstrating that malaria parasites adjust their sex ratios in response to infection genetic diversity, precisely as predicted. Theory also predicts that parasites should adjust sex allocation in response to host immunity. Whilst data are supportive, the assumptions underlying this prediction – that host immune responses have differential effects on the mating ability of males and females – have not yet been tested. Here, we combine experimental work with theoretical models in order to investigate whether the development and fertility of male and female parasites is affected by innate immune factors and develop new theory to predict how parasites' sex allocation strategies should evolve in response to the observed effects. Specifically, we demonstrate that reactive nitrogen species impair gametogenesis of males only, but reduce the fertility of both male and female gametes. In contrast, tumour necrosis factor-α does not influence gametogenesis in either sex but impairs zygote development. Therefore, our experiments demonstrate that immune factors have complex effects on each sex, ranging from reducing the ability of gametocytes to develop into gametes, to affecting the viability of offspring. We incorporate these results into theory to predict how the evolutionary trajectories of parasite sex ratio strategies are shaped by sex differences in gamete production, fertility and offspring development. We show that medical interventions targeting offspring development are more likely to be ‘evolution-proof’ than interventions directed at killing males or females. Given the drive to develop medical interventions that interfere with parasite mating, our data and theoretical models have important implications.

## Author Summary

Malaria and related parasites cause some of the most serious infectious diseases of humans, domestic animals and wildlife. To be transmitted, these parasites produce male and female sexual stages that differentiate into gametes and mate when taken up in a mosquito blood meal. Despite the need to develop a transmission-blocking intervention, remarkably little is understood about the evolution of parasite mating strategies. However, recent research demonstrates that producing the right ratio of male to female stages is central to mating success. Evolutionary theory predicts that sex ratios are adjusted in line with a variety of factors that affect mating success, including host immunity. We test this theory by investigating whether ubiquitous immune factors differentially affect the production and fertility of males and females. Our experiments demonstrate that immune factors have complex, sex-specific effects, from reducing gamete production to affecting offspring viability. We use these results to generate theory predicting how such effects shape the evolutionary trajectories of parasite sex ratio strategies. Given the drive to develop medical interventions that prevent transmission by blocking parasite mating, our results have important implications. Specifically, we suggest that medical interventions targeting offspring development are more likely to be ‘evolution-proof’ than interventions with sex-specific effects.

## Introduction

Malaria parasites are obliged to undertake a single round of sexual reproduction in the mosquito vector before they can transmit to new hosts, making this stage of their life-cycle a potential target for medical interventions [1], [2]. The success of interventions aiming to disrupt mating success will depend upon a variety of epidemiological parameters (e.g. transmission intensity/seasonality), but will also be strongly determined by the parasites' behavioural and evolutionary responses [1]–[3]. Current candidates for transmission-blocking vaccines (TBV) involve targeting proteins, expressed on the surface of sexual stages, that are essential for the fertility of males (e.g. P48/45 and P230) [4]–[8]. However, theory predicts that the efficacy of a vaccine that reduces the fertility of one sex may be eroded if parasites respond by adjusting their sex ratios in favour of the targeted sex. The study of sex allocation has been one of the most successful areas of evolutionary biology, with empirical data matching clear theoretical predictions across a variety of taxa [9]. Before describing evolutionary theory for sex allocation strategies we outline the relevant aspects of *Plasmodium* mating biology.

Every asexual replication cycle, a small proportion of parasites differentiate into male and female sexual stages – termed gametocytes – which are developmentally arrested gamete precursors [10], [11]. Gametogenesis of both sexes begins as soon as gametocytes are taken up in a mosquito blood meal, fertilization occurs within 30 minutes, and zygotes develop into the stages infective to vectors (ookinetes) after 18–20 hours [12], [13]. To differentiate into gametes, gametocytes must leave the relative safety of their red blood cells (RBCs), becoming exposed to host- and mosquito-derived factors that can block mating [12]. Males are expected to be more vulnerable than females to transmission-blocking factors due to their more complex gametogenesis and mating activities [14], [15]. Whereas female gametocytes only have to leave their RBCs to become gametes, male gametogenesis also includes three rounds of mitosis and flagellum construction to produce a (rarely achieved) maximum of eight ‘sperm-like’ gametes [16]–[20]. Mature male and female gametocytes are easily distinguished by their phenotypes as their reproductive roles result in different cellular contents [21], [22]. Mature males are terminally differentiated, only having pre-synthesized proteins and machinery for gamete production (e.g. α-tubulin II, cell cycle proteins, dynein) [11], [22], [23]. In contrast, mature female gametocytes are prepared for continued development after fertilization, having high levels of ribosomal proteins, mitochondria (which are absent in mature males) and pools of translationally repressed messenger RNAs (mRNAs; similar to P bodies in metazoan oocytes) [11], [22], [24]. Therefore, male and female gametocytes are primed for gametogenesis and zygote development, respectively [25].

Sex allocation is an important fitness-related trait in *Plasmodium* and could play an important role in the response of malaria parasites to medical interventions that aim to reduce mating success [19], [26]–[28]. Parasites could respond to transmission-blocking interventions by adjusting their sex allocation strategies via two evolutionary processes. First, if conditions within hosts are unpredictable, invariant, or if variation in within-host conditions is not a good proxy for variation in the mating conditions experienced within vectors, parasites evolve fixed (i.e. canalised) sex allocation strategies that reflect the average environment. Second, if in-host conditions reliably predict in-vector conditions, parasites will evolve to facultatively adjust their sex ratios (proportion of male gametocytes) through phenotypic plasticity. In this scenario, if asexual stage parasites detect an increase in a factor (or correlate of) that reduces mating ability in a sex-specific way, parasites will benefit from adjusting the production of male and female gametocytes in response. Given that once parasites are taken up by a vector, no further gametocyte production can occur and gametogenesis and fertilization are completed within 30 minutes, the mating environment within the blood meal is ‘imported’ from the host. Therefore, the within-host conditions will be good predictors for mating conditions and so facultative sex ratio adjustment is both predicted and observed [14].

Currently, two complementary evolutionary theories predict how and why parasites should adjust their investment into male and female gametocytes to maximise fertilization success. These theories – Fertility Insurance and Local Mate Competition – predict that parasites adjust sex ratios in response to environmental (e.g. transmission-blocking immunity) and social factors (inbreeding rate), respectively [14], [15], [29]–[34]. The ability of parasites to facultatively adjust their sex ratios in response to variation in the inbreeding rate has recently been verified [19], [27]. Additionally, data also suggest that sex ratios are altered in response to the development of immunity [19]. Host-derived immune factors make mating challenging for parasites because they can reduce and even block fertilization [35], [36]. This phenomenon, called ‘transmission-blocking immunity’ (TBI), has been extensively observed and documented across a variety of malaria parasite species [35]–[41]. The mechanisms of TBI are varied and include damaging gametocytes, preventing successful gametogenesis [36], [37], [41], [42], decreasing the ability of gametes to interact [35], [43] and preventing post-fertilization development [39], [44]. Fertility Insurance predicts that when hosts mount an immune response, the fertility of male gametocytes and/or gametes is most affected, therefore parasites should produce more males to compensate [14], [15]. Two lines of empirical data support this prediction. First, Paul *et al.* [26] showed that *P. gallinaceum* and *P. vinckei* increase their sex ratio in response to erythropoiesis, which is thought to act as a cue for the appearance of TBI factors. Second, Reece *et al.* [19] provided indirect support by suggesting that sex ratio variation observed during infections of different *P. chabaudi* genotypes is a mechanism to ensure fertility in face of within-host competition, host anaemia and TBI factors. Fertility Insurance currently provides the best explanation for the observed within-infection variation in the sex ratios of malaria parasites. However, the theory is based upon the untested assumption that TBI factors reduce the fertility of males more than females. Here we provide the first direct test of this key assumption by investigating whether reactive nitrogen species and pro-inflammatory cytokines, influence gametogenesis, gamete fertility and ookinete production.

Levels of reactive nitrogen species (RNS) and pro-inflammatory cytokines vary during malaria infections. These immune factors, which are ubiquitous components of the innate immune system, have been specifically implied in the sudden loss of infectivity to vectors that occurs during paroxysms and infection crisis [37], [41]. Specifically, tumour necrosis factor-α (TNF-α) is a potent pro-inflammatory cytokine and several studies have revealed a role for this cytokine in mediating the killing of *Plasmodium* gametocytes, across a variety of host-parasite systems [36], [41], [45]. This could occur through the stimulation of phagocytosis and nitric oxide (NO) production by white blood cells [37], [46], [47], as these are capable of phagocytosing opsonized gametes in the mosquito midgut [48] and the inhibition of NO synthesis by white blood cells reduces in 60% the inactivation of *P. falciparum* and *P. vivax* gametocytes [37], [49]. NO is produced by the enzyme inducible nitric oxide synthase in response to infection, in both hosts and vectors, and is extremely toxic at high doses. NO is a highly reactive molecule, thus a significant extent of the damage it causes is indirect, through the production of RNS (such as peroxynitrite, nitrates, nitrites or S-nitrosothiols) that frequently function as the ultimate effectors [50]. Hereafter, unless otherwise stated, we use the term ‘RNS’ to refer to NO and its reaction products. During *Plasmodium* infections, RNS appears to impair asexual replication, gametogenesis and zygote development [37], [42], [44], [51]. Levels of RNS increase during *P. yoelii* infections and reduce ookinete production when either gametocytes or gametes are exposed [42]. Furthermore, RNS have been shown to induce the programmed cell death of *P. berghei* ookinetes [52] and to extensively reduce *P. berghei* oocyst burdens in *Anopheles* mosquitoes [44]. This is, at least in part, the result of a pro-inflammatory response, in which host cytokines induce the mosquito to increase NO (and therefore RNS) production [53].

**Figure 1:**
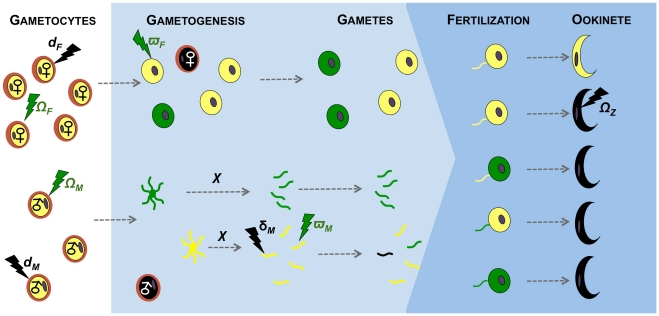
Effects of immunity on gametogenesis and fertility of malaria parasites. The effects of transmission-blocking immune factors on the sexual development of malaria parasites investigated in our model. Female and male gametocytes circulating in the host (white background) undergo gametogenesis when taken up by a mosquito vector (blue background). Each male gametocyte differentiates into *χ* gametes (*χ*≤8) and each female gametocyte produces one gamete. Male gametes locate and fertilise female gametes, and the resulting zygotes develop into ookinetes. Immune factors circulating in the host can act on males and females throughout their sexual development, from gametocytes to zygotes. The developmental stages of females are shown above the stages of males and each individual gametocyte/gamete is shown in the same relative position throughout development. The effects of immune factors (lighting) on sexual stages can either be cryptic (i.e. render gametocytes/gametes dysfunctional; green), or fatal (i.e. gametocytes/gametes die; black). Healthy, unaffected, parasites are represented in yellow, dysfunctional parasites in green, and dead parasites in black. Immune factors kill female gametocytes with probability *d_F_* and male gametocytes or gametes with probabilities *d_M_* or *δ_M_*, respectively. Dead sexual stages do not participate further in the mating pool. Immune factors render female gametocytes and gametes dysfunctional with probabilities *Ω_F_* and *ϖ_F_* respectively, and male gametocytes and gametes with probabilities *Ω_M_* and *ϖ_M_*, respectively. Dysfunctional gametocytes/gametes participate in the mating pool and can be fertilized as for healthy gametes, however zygotes are unviable and die before reaching the ookinete stage. Immune factors can also directly lead to zygote death with probability *Ω_Z_*. All possible fertilization scenarios are represented: mating between two healthy gametes, mating between one healthy and one dysfunctional gamete and mating between two dysfunctional gametes.

Here, we use the rodent malaria parasite *Plasmodium berghei* to conduct a series of experiments to investigate how RNS and TNF-α influence mating success and ookinete production and develop theoretical models that predict the evolution of sex allocation strategies, given the effects observed in our experiments. Therefore, we use these immune manipulations as ‘proof-of-principle’ for other factors with similar effects on the sexual reproduction and transmission of malaria parasites. Specifically, we test whether: (1) RNS and TNF-α have dose dependent effects on male gametogenesis (exflagellation) and ookinete production; (2) exposure of male and female gametocytes to both RNS and TNF-α influences their sexual development; (3) the greater effect of RNS we observe on male gametogenesis results in sex-specific fertility effects; and (4) the observed effects of RNS depend on the developmental stage at which parasites are exposed. Our results reveal that RNS reduces male but not female gametogenesis and impairs the fertility of both sexes, whereas TNF-α only affects zygote development. The relative importance of reduced gametogenesis, impaired mating ability and reduced post-mating development have not been explicitly considered by Fertility Insurance theory. Therefore we develop a new mathematical model to derive predictions for how the effects of immune factors generated naturally or by a medical intervention are likely to impact upon parasite sex ratio evolution (a schematic of the biological effects included in the model is presented in Figure 1).

## Results

All the experiments we describe below were performed *in vitro*, using gametocytes harvested form *Plasmodium berghei* infected mice. Parasites were either cultured in conditions that ‘mimicked the vector’ (in which they immediately became activated and underwent gametogenesis and mating; media at pH 8 and 21°C), or conditions that ‘mimicked the host’ (in which gametocytes remained developmentally arrested; pH 7.25, 37°C) [19]. Parasites cultured in host mimicking conditions became activated and underwent gametogenesis if subsequently exposed to vector mimicking conditions. We manipulated exposure to TNF-α with recombinant mouse TNF-α and RNS exposure with L-ana (L-Arginine p-nitroanilide dihydrochloride) and SIN-1 (3-morpholinosydnonimine hydrochloride). L-ana is an inhibitor of NO synthesis and SIN-1 donates RNS in solution (see methods for details) [54]. We exposed parasites to RNS and TNF-α treatments in 1 ml cultures with 15 or 20 µl parasitized blood.

### Experiment 1: Dose-dependent effects of RNS and TNF-α

We first tested whether RNS and TNF-α influence sexual reproduction by exposing parasites to different concentrations of these factors and assaying exflagellation and ookinete production. We incubated parasites in vector mimicking media across seven concentrations of SIN-1 (ranging from 0 to 1 mg/ml) [55] and five concentrations of recombinant mouse TNF-α (from 0 to 1 µg/ml; see Methods). Increasing concentrations of SIN-1 caused a significant linear decrease in the densities of exflagellating males (F_(1,35)_ = 16.28, P<0.0001; transformed y = 0.16-0.10x) and ookinetes (F_(1,35)_ = 25.86, P<0.0001; transformed y = 0.17-0.18x). Similarly, TNF-α also caused a significant linear decrease in the densities of exflagellating males (F_(1,15)_ = 6.83, P = 0.012; y = 0.23-0.09x) and ookinetes (F_(1,15)_ = 17.53, P<0.0001 ; transformed y = 0.54-0.37x).

### Experiment 2: Effects of RNS and TNF-α on gametogenesis and ookinete production

Having found significant negative effects of RNS and TNF-α on exflagellation and ookinete production we investigated whether these factors interacted with each other to further reduce parasite mating success and if these effects depended on the developmental stage at which parasites were exposed (i.e. in host or vector conditions). For this set of experiments we used a fully cross-factored design, consisting of two RNS and two TNF-α levels (see Methods).

**Figure 2:**
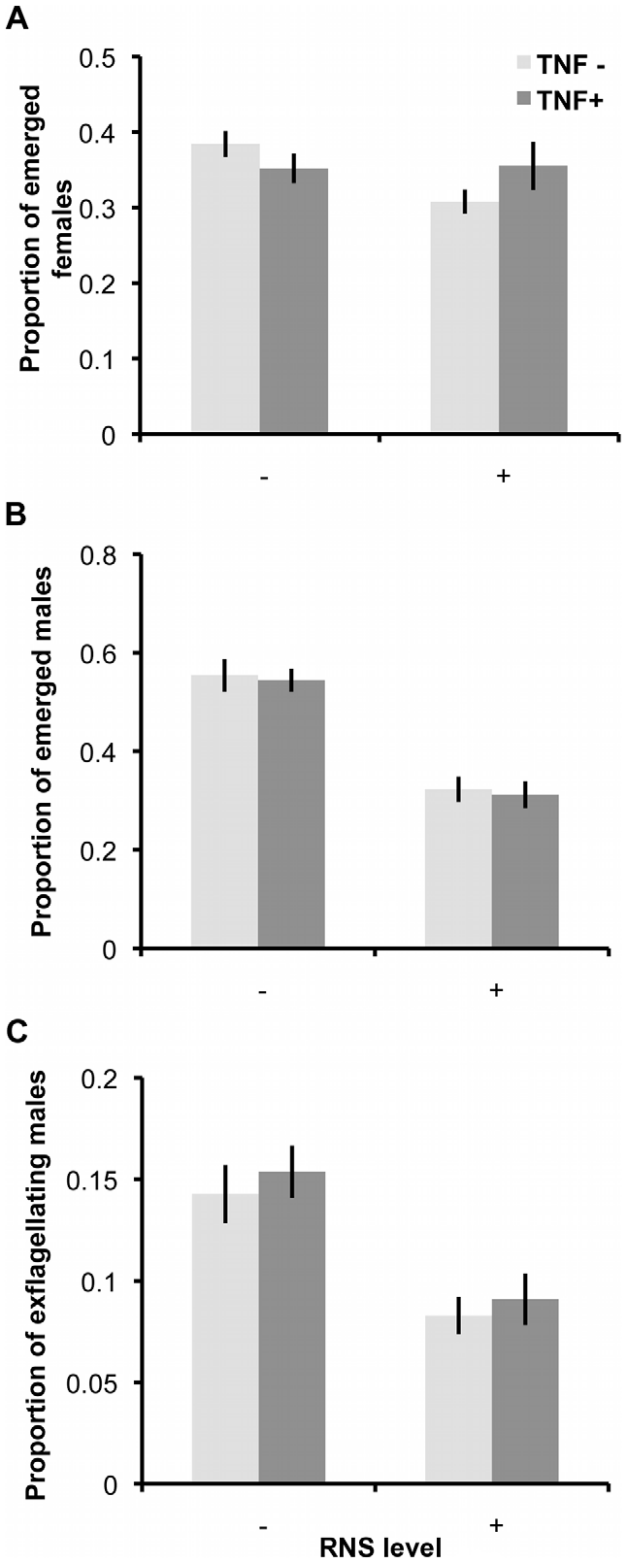
Ability of gametocytes to undergo gametogenesis after exposure to RNS and TNF-α. Mean (± S.E.) proportion (n = 20) of emerged female gametes (A), emerged male gametocytes (B), and exflagellating male gametes (C), relative to the total number of male or female gametocytes/gametes observed, when gametocytes are exposed to immune factors during incubation in ‘host conditions’ and then activated in un-manipulated ‘vector conditions’ media.

First, we investigated the effects of RNS and TNF-α on gametocytes by incubating parasites for 60 minutes in host mimicking media. We then replaced treatment media with vector mimicking media (without RNS or TNF-α manipulations) to stimulate gametogenesis and quantified the development of male and female gametocytes into gametes using the following classifications: (a) mature gametocytes still inside their RBC, (b) gametocytes that had emerged from the RBC and (c) exflagellating male gametes (see Methods for criteria). We present the proportion of a given developmental stage relative to the total number of observed gametocytes/gametes of the same sex (Figure 2). The proportion of emerged female gametocytes was not significantly influenced by either RNS (χ^2^_1_ = 2.72, P = 0.099) or TNF-α (χ^2^_1_ = 0.12, P = 0.731; or their interaction χ^2^_1_ = 3.38, P = 0.066). In contrast, the proportion of male gametocytes that emerged from RBCs was significantly reduced by RNS (F_(1, 59)_ = 81.29; P<0.0001; mean ‘RNS−’ 0.55±0.02; ‘RNS+’ 0.32±0.02) but not by TNF-α (χ^2^_1_ = 0.16, P = 0.689; or their interaction χ^2^_1_<0.01, P = 0.982). Similarly, the ability of males to exflagellate was significantly reduced by RNS (F_(1, 59)_ = 33.40; P<0.0001; mean ‘RNS−’ 0.15±0.01; ‘RNS+’ 0.09±0.01) but not by TNF-α (χ^2^_1_ = 0.85, P = 0.36; or their interaction χ^2^_1_ = 0.02, P = 0.885).

**Figure 3:**
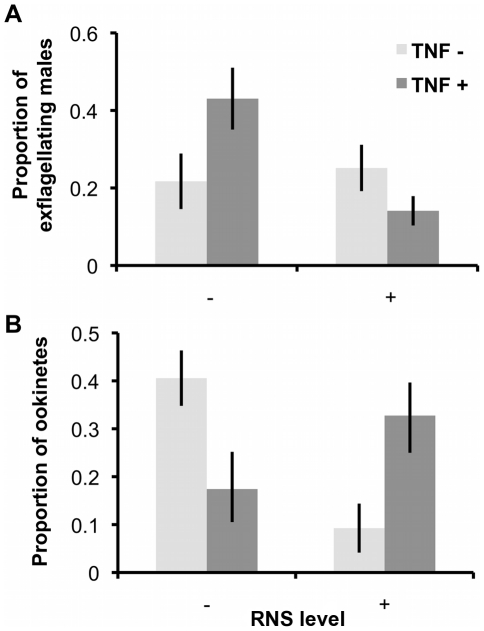
Exflagellation rates and ookinete production after exposure to RNS and TNF-α during gametogenesis. Mean (± S.E.) proportion of exflagellating male gametes (A; n = 16) or ookinetes (B; n = 9) produced when parasites are exposed to RNS and TNF-α during gametogenesis (in-vector conditions media). Proportions are relative to the total number of exflagellating male gametes or ookinetes produced from each infection, across treatments.

Second, we investigated the effects of RNS and TNF-α on exflagellation and ookinete production by incubating parasites in culture media mimicking the vector environment (Figure 3). In line with the results from our previous experiments, the proportion of exflagellating males was significantly reduced by RNS (F_(1, 45)_ = 11.24, P = 0.002; mean ‘RNS−’ 0.32±0.06; ‘RNS+’ 0.12±0.03). This effect was enhanced by TNF-α (interaction: F_(1, 45)_ = 6.67, P = 0.014) but in the absence of RNS, TNF-α had no significant effect (F_(1, 45)_ = 1.90, P = 0.175). Conversely, the effect of RNS and TNF-α on ookinete production depended on each others presence (interaction F_(1, 24)_ = 14.91, P = 0.001). Specifically, ookinete production was reduced by TNF-α but only in the absence of RNS (mean ‘TNF-α−’ 0.41±0.06; ‘TNF-α+’ 0.17±0.07), whereas RNS reduced ookinete production but only when TNF-α was absent (mean ‘RNS−’ 0.41±0.06; ‘RNS+’ 0.09±0.05).

### Experiment 3: Sex-specific effects of RNS on fertility

**Figure 4:**
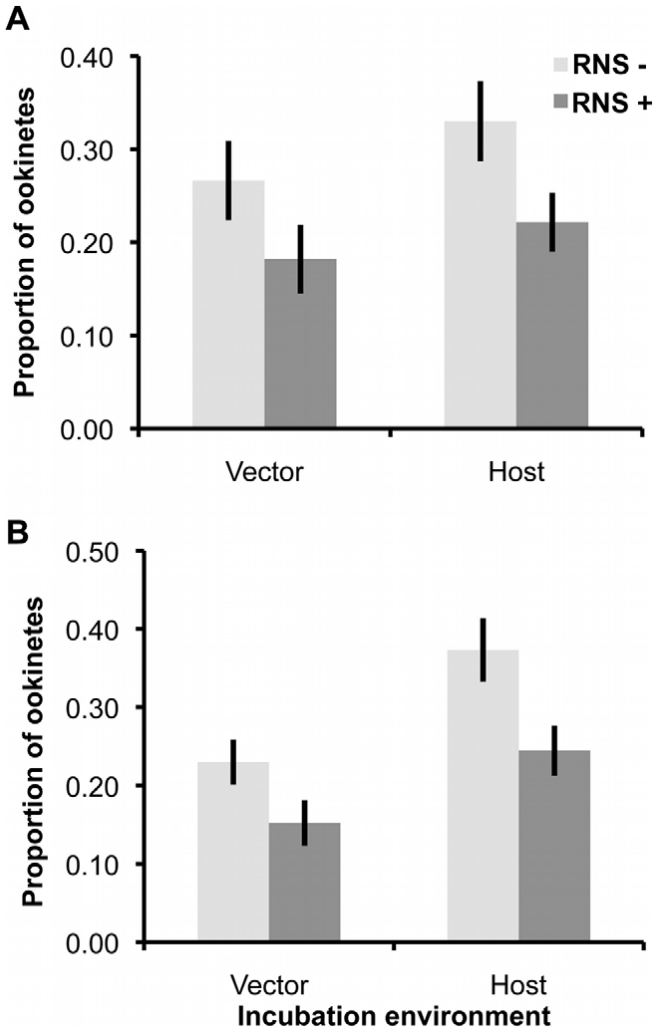
Ookinete production after exposure of males or females to RNS, before or during gametogenesis. Mean (± S.E.) proportion (n = 19) of ookinetes produced, when females (A) or males (B) are exposed to RNS as gametocytes (in-host conditions media) or during gametogenesis (in-vector conditions media). Proportions are relative to the total number of ookinetes produced by the focal sex from each pair of infections.

Experiment 2 revealed that only RNS had a significant effect on gametogenesis, in which male but not female development was impaired. Therefore, we tested whether these effects translated into sex-specific differences in fertility (i.e. whether matings with RNS exposed gametocytes/gametes resulted in fewer ookinetes), when parasites were exposed as gametocytes (in host-mimicking media) or during gametogenesis (in vector-mimicking media). We separately exposed each sex to RNS using two genetically transformed (knock-out; KO) *P. berghei* lines: Pbs48/45ko and Pbs47ko [4], [6], [22], which produce unviable male and female gametes, respectively. This allowed us to assay the fertility consequences of exposing one sex to RNS by providing exposed parasites with a surplus of unexposed mates from the opposite sex and assaying ookinete production (Figure 4).

We observed that RNS exposure significantly reduced fertility of both males and females regardless of whether parasites were exposed as gametocytes or during gametogenesis (F_(1,131)_ = 15.87, P = 0.0001; mean ‘RNS−’ 0.30±0.02; ‘RNS+’ 0.20±0.02). In contrast to our predictions, RNS did not have sex-specific effects (treatment:sex interaction: χ^2^_1_ = 0.023, P = 0.88), nor was this effect influenced by exposing parasites to RNS in host- or vector-mimicking environments (treatment:environment interaction: χ^2^_1_ = 0.366, P = 0.55). However, across all treatments, parasites exposed in host conditions produced significantly more ookinetes than those exposed in vector conditions (F_(1,131)_ = 10.19, P = 0.0018; mean ‘Host’ 0.29±0.02; ‘Vector’ 0.21±0.02).

### Theoretical model

We incorporate our experimental results into Fertility Insurance theory by developing a mathematical model to explore the impact of transmission-blocking factors on the evolution of parasite sex allocation strategies. Specifically, we examine whether sex ratio adjustment could compensate for transmission-blocking factors with the following effects on males or females: preventing male or female gametocytes from undergoing gametogenesis (as each female gametocyte only produces one gamete, killing of these stages is mathematically equivalent); blocking the mating ability of male gametes; and causing damage to gametocytes or gametes such that mating can occur but zygotes are not viable. We term the latter phenomenon, of cryptic damage to gametocytes or gametes that results in a dead zygote, as dysfunction. Note that, although we do not observe all of the effects on all stages and all sexes, we incorporate them all in the model (illustrated in Figure 1), as they are theoretical possibilities. Also, our model makes no assumptions about whether parasites evolve fixed (i.e. canalised) or facultative (i.e. plastic) sex allocation strategies.

First, we show that all zygote mortality effects (i.e. treatments leading to 0<*p*<1) have no impact on the evolutionarily stable (ES) sex ratio [56], [57]. We write *W = ζ*(*z*) *p*, i.e. fitness is the product of zygote production and zygote viability, where zygote production depends upon sex ratio but zygote viability does not. The direction of selection is given by the derivative of fitness with respect to sex ratio [58], and this ‘marginal fitness’ is d*W*/d*z* = (d*ζ*/d*z*)*p*. The ES sex ratio z* satisfies d*W*/d*z*|_z = z*_ = 0, i.e. selection does not favour an increase or decrease in sex ratio when the population is at the ES sex ratio, and this is equivalent to the condition d*ζ*/d*z*|_z = z*_ = 0 for all *p*>0. Since ζ is not a function of *p*, it follows that z* is not a function of *p* (and hence is not a function of *Ω_Z_*, *Ω_M_*, *Ω_F_*, ϖ_M_ or ϖ_F_; see Methods and Figure 1 for symbol definitions). Therefore, treatments that simply impact upon the viability of zygotes (e.g. cause gametocyte/gamete dysfunction) are not expected to have an evolutionary impact upon parasite sex ratios.

**Figure 5:**
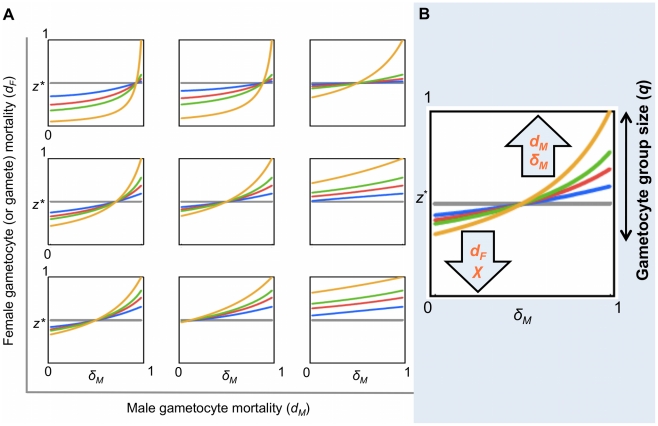
Evolutionarily stable sex allocation strategies when sex- and stage-specific mortality rates vary. Effect of male and female gametocyte mortality and male gamete mortality on the ES gametocyte sex ratio (z*), for a clonal population, when the number of gametes produced per male gametocyte (*χ*) is 2 (this fecundity has been estimated for this system by other studies; see ref. [19]). Figures S1, S2, S3 show similar patterns to Figure 5A for *χ* = 1; 4; 8, respectively. (A) For each plot within the panel, z* varies with male gamete mortality rate (*δ_M_*). The coloured lines represent different gametocyte group sizes (*q*): 2 (grey), 5 (blue), 10 (red), 20 (green) and ∞ (yellow). Each plot depicts different parameter combinations of male gametocyte (*d_M_* = 0.1; 0.5; 0.9) and female mortality rate (*d_F_* = 0.1; 0.5; 0.9), with *d_M_* increasing left to right and *d_F_* increasing bottom to top. (B) Cartoon summarizing the effects observed in Figures 5A and S1, S2, S3. The set of possible values for z* is strongly influenced by *q*. The number of gametes of each sex reaching the mating pool (which depends on the mortality parameters and on *χ*) influences z* within the constraints determined by *q*. Within each plot, the effects of *δ_M_* and *q* on z* can be clearly observed: the magnitude of sex ratio change increases with *q* and z* increases to compensate for higher *δ_M_*. The effects of *d_M_* and *d_F_* can be observed by comparing the points where the lines cross the y axes (i.e. *δ_M_* = 0) across the plots: z* increases along rows with increasing *d_M_* and decreases up the columns with increasing *d_F_*. The effect of *χ* on z* can be observed by comparing plots that are in the same position in different figures: sex ratio becomes more female biased as *χ* increases.

Second, to investigate the impact of model parameters arising from gametocyte or gamete killing on the ES sex ratio, we write an explicit expression for expected fitness:
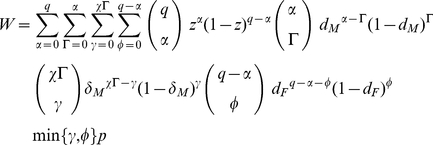
(1)The condition d*W*/d*z*|*_z_*_ = z*_ = 0 can be solved numerically for z* for any numerical parameter set (*q*, *d_M_*, *d_F_*, *δ_M_*). An exploration of the ES sex ratio z* across this parameter space is presented in Figures 5 and S1, S2, S3. Specifically, we recover the prediction that the gametocyte ES sex ratio will be biased towards the more limiting sex when factors prevent male or female gametocytes from undergoing gametogenesis or block the mating ability of male gametes.

## Discussion

Evolutionary theory developed to explain the sex allocation strategies of metazoan taxa has enjoyed huge success. Recently, there has been growing interest in whether this theory could be applied to protozoans, particularly malaria parasites [14]. The sex ratios of malaria parasites are normally female biased, but extensive variation occurs during the course of infections [27]. Evolutionary theory offers an explanation for this variation and predicts that in-host conditions will influence parasite sex allocation strategies if host-derived immune factors disproportionately reduce the fertility of males relative to females [14], [15], [29]–[34]. Here, we tested this assumption by quantifying the effects of two well-known innate TBI factors (RNS and TNF-α) on sexual development and fertility of malaria parasites [15], [31]. We show that: (1) RNS and TNF-α reduce the densities of exflagellating males and ookinetes in a dose-dependent manner; (2) TNF-α can reduce ookinete densities, but only in the absence of RNS (Figure 3); (3) RNS impairs male but not female gametogenesis (Figure 2 and 3), and reduces the fertility of both males and females independently of whether parasites are exposed as gametocytes or during gametogenesis (Figure 4). We then explored the consequences of our results for parasite sex ratio evolution, by incorporating them into Fertility Insurance theory (Figures 1 and 5) [15], [31]. Specifically, our model demonstrates that the ES sex ratio will be biased towards the sex that has a lower number of surviving gametes reaching the mating pool and that the extent of this bias increases as the number of gametocytes in the mating group (*q*) increases. We also show that factors causing gametes to become dysfunctional (resulting in inviable zygotes) do not affect the ES sex ratio. Below, we discuss the results of our experiments, explain the evolutionary predictions of our model and its implications for the development of transmission-blocking interventions.

### RNS, TNF-α and the sexual development of malaria parasites

In our experiments, RNS reduced male but not female gametogenesis while impairing the fertility of both sexes. How can these results be explained? In parasitic infections, high levels of RNS may cause: oxidative damage of DNA (leading to mutations and strand brakes); inhibition of DNA repair and synthesis; inhibition of protein synthesis; inhibition of mitochondrial activity; down- or up-regulation of cytokine (e.g. TNF-α) levels [50], [59]. As described in the introduction, male and female gametocytes are prepared for gametogenesis and zygote development respectively [25]. If RNS can impair DNA synthesis and/or microtubule assembly, males would not be able to produce gametes. In contrast, female gametogenesis does not involve these activities and females ‘simply’ need to leave their RBCs, for which they use the contents of pre-synthesized secretory organelles called osmiophilic bodies [60]. Therefore, whilst female gametogenesis and mating *per se* is unlikely to be influenced by RNS, the development of fertilized females into zygotes and ookinetes is likely to be affected. For example, damage to stored mRNA and inhibition of protein synthesis or mitochondrial activity (e.g. cytochrome oxidases) would impair meiosis (at ∼3 h after fertilization) and zygote development, but not impair fertilization [18], [50], [59]. These effects could explain the observed results, because instead of reducing the ability of females to differentiate into gametes, the effects of RNS would be expressed after fertilization (which we term dysfunction) and lead to female-dependent zygote death, resulting in fewer ookinetes. Here we did not identify the causal RNS and their relative contributions. However, this will be important if transmission-blocking interventions cause or mimic the activities of RNS.

Our experiments show that TNF-α consistently reduces ookinete production and whilst we observed a reduction in exflagellation in some experiments, this effect was inconsistent. Why does TNF-α reduce ookinete production? As TNF-α functions are mainly modulatory and need time to develop, it is possible that gametogenesis and mating occur before the effects of TNF-α manifest. Ookinete development takes about 18–20 hours from fertilization and during this time TNF-α could exert its effects, which could also involve the activation of apoptotic-like death [61], [62]. Recent experiments provide support for our interpretations, as the deletion of genes coding for proteins essential for the storage and stabilization of translationally repressed mRNAs, in female gametocytes/gametes, do not reduce fertilization success, but substantially reduce the differentiation of zygotes into ookinetes [24], [63]. Interestingly, deletion of different genes can affect zygotes throughout development, suggesting that damage to stored mRNA could abort zygote development at multiple stages (e.g. before or after meiosis) [24].

### Evolution of parasite sex allocation strategies: Theoretical predictions

The results of our experiments show that TBI factors can affect the sexual development and fertility of male and female parasites and that the stage at which this occurs is sex-specific. As illustrated in Figure 1, we incorporated the observed and potential effects of transmission-blocking factors on males and females, at all stages of development, into Fertility Insurance theory and generated new predictions for the evolution of parasite sex allocation strategies. Our model predicts that the ES gametocyte sex ratio will be insensitive to variation in gametocyte or gamete dysfunction and zygote mortality. This means that treatments that impact upon the viability of zygotes are not expected to have an evolutionary impact upon parasite sex ratios. In contrast, we predict that the best (ES) sex ratio strategy will vary depending on an interaction between gametocyte group size (*q*), number of gametes formed per male gametocyte (0≤χ≤8) and gamete and/or gametocyte mortality. Although, our model makes no assumptions about whether parasites achieve an ES sex ratio through the evolution of facultative or fixed sex allocation strategies, facultative sex allocation is predicted for reasons already outlined in the introduction.

In the context of clonal infections, the ES sex ratio maximises the expected number of viable zygotes, i.e. maximises the expected number of gametes of the minority sex present in the mating pool (this excludes dead gametocytes/gametes, but includes dysfunctional gametocytes/gametes). For an infinite gametocyte group size (i.e. *q*→∞), that behaves deterministically, the ES sex ratio is one that leads to the same number of male and female gametes being present in the mating pool. This is the sex ratio z* that satisfies cz* = 1-z*, i.e. z* = 1/(*c*+1), where *c* is the number of male gametes, able to mate, produced per male gametocyte [15], [32]. Thus, the ES sex ratio is female biased if *c*>1, and male biased if *c*<1 (Figures 5 and S1, S2, S3). However, for finite mating groups (*q*<∞) – that behave stochastically – the expectation of mating success must be calculated over the whole distribution of possible outcomes. This will tend to reduce the extent to which the sex ratio is biased towards the sex favoured in the deterministic case [15], [31]. For example, in the extreme of a gametocyte group size of two (*q* = 2; the lowest mating group size for which mating success is possible), the ES sex ratio is always z* = 0.5 (regardless of other parameter values), to maximise the probability of both sexes being present (Figures 5 and S1, S2, S3). Additionally, we reveal that, in a small portion of parameter space – corresponding to very small gametocyte group sizes, low female mortality, and high male gametocyte mortality and fecundity (*χ*) – fertility insurance can even lead to a sex ratio bias in the opposite direction (i.e. producing a female biased sex ratio, despite the risk of the absence of males in the mating pool; Figures S2 and S3). This non-intuitive result is due to the way stochastic variation in the number of gametocytes of each sex alters the variance as well as the expected number of gametes of each sex that reach the mating pool. Although the conditions under which this occurs are restrictive, they may be met in natural infections, as many individuals carry gametocytes at extremely low densities [64]. In the context of our experiments and assuming parasites can facultatively adjust sex ratios, our model predicts that if *q* is high enough to allow for sex ratio adjustment, then RNS should induce parasites to increase the production of male gametocytes.

Our data suggest that RNS reduced female fertility by rendering gametocyte/gametes dysfunctional, so that their fertilisation results in the production of unviable zygotes. The reduction in ookinete production by TNF-α could also be due to male or female dysfunction or, more likely, through increasing zygote mortality. Therefore, we examined the influence of gametocyte and gamete dysfunction and zygote mortality on the evolution of parasite sex allocation strategies. We found that the ES gametocyte sex ratio is independent of these factors (i.e. the occurrence of gametocyte/gamete dysfunction and zygote mortality does not change the relative fitness of different sex ratio strategies). Put simply, this suggests that zygote mortality or gametocyte/gamete dysfunction will not impose selection on parasite sex allocation strategies as parasites cannot compensate for the loss of reproductive success through sex ratio adjustment. More broadly, other immune factors, such as antibodies or complement, could also impair the sexual reproduction of malaria parasites and the effects of such factors should be easily interpreted in light of our theoretical models.

To bring our mathematical modelling in line with our experiments we have focused on the importance of mortality and dysfunction throughout the sexual development of malaria parasites. However two additional factors have an important impact in sex allocation strategies of malaria parasites: (1) the inbreeding rate and (2) the rate at which asexually replicating parasites commit to gametocyte production (conversion rate). The effect of inbreeding on the ES sex ratio is well understood, with theory (Local Mate Competition) enjoying strong empirical support [14], [19], [29], [32]–[34]. For clonal mating groups, the ES sex ratio strategy is the one that maximises the overall mating success of the infection as the parasites behave as a single, unified decision maker [14], [27]. In contrast, in mixed infections, conflicts between clones occur, such that the ES sex ratio is the one that maximises each individual clone's inclusive fitness and not the overall mating success of the infection [14], [27]. But for the work we present here, extending our model to allow for a finite number of independent clones per host would not change the qualitative results we present. Fertility Insurance theory predicts that if a low conversion rate results in a small number of gametocytes being taken up by the vector (i.e. small *q*), parasites should produce a less female biased sex ratio than expected by the inbreeding rate alone. This is due to the stochastic risk of too few males being present in the blood meal to fertilize the females when sex ratios are female biased [15]. One intuitive solution for this would be to produce more gametocytes. However, given that gametocyte production comes at a cost to asexual replication, parasites face a trade-off between investment in in-host survival and reproduction (i.e. transmission). Increasing gametocyte conversion is a solution that will not always be available and might be impossible when parasites are ‘stressed’ (e.g. by in-host competition and low doses of anti-malarial drugs) [65], [66]. Therefore, if transmission-blocking interventions also affect asexual stages and reduce in-host survival, parasites are likely to reduce conversion rates and produce fewer gametocytes.

### Implications for transmission blocking interventions

Our model reveals that an intervention with a sex-specific effect on mating ability will elicit an evolutionary response. However, sex ratio adjustment cannot fully rescue zygote production, given that an increase in the number of male gametocytes comes at the cost of decreasing the number of female gametocytes. Nevertheless, in a scenario of widespread transmission-blocking vaccination or treatment with gametocidal drugs with a sex-specific effect, natural selection will “compare” the fitness of parasites that do, and do not, adjust their sex allocation strategies, leading to an increase in the frequencies of the former. Therefore, quantifying the impact of sex ratio adjustment on rescuing fertility and thus, fitness is now required. In contrast, our model also reveals that a transmission-blocking factor resulting in zygote mortality or gametocyte/gamete dysfunction will be ‘evolution proof’ with respect to parasite sex allocation strategies. Therefore, we suggest that current efforts to prevent fertilization by targeting proteins with sex-specific phenotypes, such as P230, P48/45 (involved in gamete attachment) or Pfg377 (female emergence from the RBC), will be less effective than vaccines targeting zygote development (e.g. P28) [5], [60], [67]. An alternative transmission-blocking approach could cause dysfunctional female gametes by targeting the expression of female-specific translationally repressed mRNAs [24]. Furthermore, a transmission-blocking intervention combining targets for gamete dysfunction and zygote death would minimize possible redundancy effects, which have been observed in several knock-outs of malaria parasites (e.g. P48/45) [6].

### Conclusions

Given the drive to develop transmission-blocking interventions that disrupt sexual reproduction in malaria parasites, there is an urgent need to evaluate how their short- and long-term success will be influenced by parasite mating strategies. Here, we combined experiments with mathematical modelling to predict how transmission-blocking factors influence parasite sex allocation strategies. Our model predicts that transmission-blocking interventions causing gametocyte/gamete dysfunction and/or zygote mortality will be ‘evolution-proof’ from the perspective of imposing selection on parasite sex ratio strategies, i.e. parasites may still evolve other strategies or traits to cope with a transmission-blocking intervention, but these will have to be independent of sex allocation. Put simply, understanding the behavioural strategies that parasites have evolved to cope with naturally occurring transmission-blocking immune factors, will inform predictions for how they will respond to a transmission-blocking factor. More broadly, understanding how, when and why parasites respond to changes in their in-host environment will facilitate the development of interventions that induce parasites to make decisions that are suboptimal for their transmission success, but that are clinically or epidemiologically beneficial. For efficient progress, synergy between research directed at evolutionary and mechanistic explanations for parasite traits and strategies is required.

## Methods

### Hosts and parasites

We maintained MF1 mice, aged 8–10 weeks (Harlan-Olac, UK; or in house supplier, University of Edinburgh), on *ad libitum* food (RM3(P), DBM Scotland Ltd, UK) and water (supplemented with 0.05% PABA to enhance parasite growth), with a 12 hour light cycle, at 21°C. We initiated infections by intra-peritoneal inoculation of 10^7^ parasitized RBCs in 100 µl carrier consisting of 50% Ringers (27 mM KCl, 27 mM CaCl_2_, 0.15 M NaCl), 47.5% heat-inactivated foetal bovine serum and 2.5% heparin (5 units ml^−1^). For experiments 1 and 2, we inoculated female mice, previously (day −3 or −4) treated with 60 mg/kg of phenylhydrazine (PHZ), with *P. berghei* line 820 [68]. For experiment 3 we inoculated male mice (PHZ treatment: 125 mg/Kg, day −2) with one of two *P. berghei* KO lines: Pbs48/45ko or Pbs47ko [4], [6], [22]. We treated mice with PHZ because the resulting release of young RBCs increases gametocyte production in *P. berghei*, which maximises the number of gametocytes that can be harvested for *in vitro* mating experiments [69]. For each experiment, parasites were collected from mice on day 3 or 4 post-infection, and each infection contributed parasites to all treatments to control for any potentially confounding influences of differences between infections.

### Animal ethics statement

All the protocols involving mice passed an ethical review process and were approved by the U.K. Home Office (Project License 60/3481). Work was carried according to the Animals (Scientific Procedures) Act, 1986.

### Culture conditions

In order to manipulate the levels of RNS and TNF-α we used the following chemicals: recombinant mouse TNF-α (Sigma, UK), L-ana (Sigma, UK) and SIN-1 (Sigma, UK). We dissolved all chemicals in phospate buffered saline and exposed parasites to treatments in 1 ml cultures with 15 or 20 µl parasitized blood. L-ana is a specific inhibitor of the activity of the enzyme inducible nitric oxide synthase which becomes active in response to infection. SIN-1 donates NO and/or superoxide, in solution, at different rates depending on the specific conditions in which SIN-1 is incubated [54], [70], [71]. However, given that superoxide and NO react with each other at an extremely fast rate to produce peroxynitrite (ONOO^−^), SIN-1 is likely to act as a donor of either NO or peroxynitrite, depending on the rates at which SIN-1 generates NO and superoxide [54]. The oxygen concentration of the solution is one of the major determinants of whether SIN-1 behaves as a NO or peroxynitrite donor, donating mostly NO in anaerobic conditions and peroxynitrite in aerobic conditions [54]. In our cultures, oxygen concentrations were in-between fully anaerobic and aerobic conditions, as parasites were incubated in closed 1.5 ml tubes. Biological agents, such as human plasma or heme proteins, which are similar to components of our cultures (e.g. mouse plasma, haemoglobin) increase the capacity of SIN-1 to donate NO [54]. Furthermore, as peroxynitrite can react to produce several RNS (e.g. nitrite, nitrate, S nitrosothiols or nitrosyl-metal complexes) and as we did not measure the specific contributions of each of these factors, we use the term RNS to refer to the factors present in cultures exposed to SIN-1 [50], [61], [72]. We did not measure RNS and TNF-α levels in our cultures for three reasons. First, our focus is on testing the effects of RNS and TNF-α on the sexual development of parasites. As our experiments were designed so that each host contributed blood and parasites to all treatment groups in a given experiment, this controls for any variation between infections and ensures that our results are due to the RNS and TNF-α manipulations each culture was subjected to. Second, TNF-α levels were directly manipulated with recombinant mouse TNF-α. Third, we are not aware of any method that would allow us to measure total levels of the different RNS in small volume cultures.

### Experiment 1

We set up cultures with vector mimicking media for the following SIN-1 concentrations: 0, 0.00001, 0.0001, 0.001, 0.01, 0.1 and 1 mg/ml [55], with 6 mice contributing parasites to each treatment. We tested the following concentrations of recombinant mouse TNF-α: 0, 0.005, 0.01, 0.5 and 1 µg/ml with 4 mice contributing parasites to each treatment. We recorded the densities of exflagellating males after 15–20 minutes and ookinetes after 18–20 hours using a haemocytometer.

### Experiment 2

We used the following RNS and TNF-α levels: 1 mg/ml SIN-1 (RNS+), 1 mg/ml of L-ana (RNS−), and presence (TNF-α+) or absence (TNF-α−) of 1 µg/ml recombinant mouse TNF-α. Parasites from each of 20 mice were exposed to all four combinations of treatments. We used the following criteria to classify developmental stages of gametogenesis after 15 minutes incubation in vector mimicking media: (1) Mature gametocytes: still inside their RBC; females have blue-purple cytoplasm, small, well defined purple nucleus surrounded by a small nucleolus; males have pink-yellow cytoplasm and large disperse pale-pink nucleus. (2) Emerged females: female gamete condensed into a more circular shape, without a vacuole, cytoplasm staining a more intense blue and a less obvious nucleolus than in a female gametocyte. (3) Emerged male: male gamete with a large circular nucleus in the centre of the cell surrounded by a ring of cytoplasm. (4) Exflagellating male: emerged male gamete progressed to forming up to 8 flagella that protrude from the cell and stain red-purple [73]–[75]. We also recorded the densities of exflagellating males and ookinetes as described for experiment 1.

### Experiment 3

We infected 38 mice with Pbs47ko (n = 19) or Pbs48/45ko (n = 19). We set up mating cultures following Reece et al. [19], by pairing infections according to proximity of their sex ratios, calculated from the densities of Pbs48/45ko female gametocytes in giemsa stained smears (using criteria described for Experiment 2) and Pbs47ko exflagellating males (as for Experiment 1). To avoid pseudo-replication, each infection was only used in 1 pair. For each pair of mice, we made 8 sets of 1 ml cultures, either with (RNS+) or without (RNS−) 1×10^−5^ mg/ml SIN-1, mimicking host (60 min. incubation) or vector conditions (15 min. incubation), to which we added 15 µl of parasites from one of the infections in each pair. These single sex cultures provided ‘exposed’ parasites for fertility testing, and corresponded to the following factorial design: 2 conditions (host/vector)×2 SIN-1 exposures (RNS+/−)×2 sexes (male/female). After incubation we replaced media in all cultures with 1 ml vector mimicking media (without any SIN-1 manipulation). While ‘exposed’ parasites were incubating, we collected 60 µl of blood from each infection's pair and added these ‘unexposed’ parasites to 4 ml cultures in vector mimicking media (without SIN-1). Each 1 ml culture of the ‘exposed’ parasites was then added to a 4 ml culture containing its ‘unexposed’ pair and incubated to produce ookinetes (as for Experiment 1). This allowed us to ensure that the mating success of the ‘exposed’ sex would not be limited by the availability of ‘unexposed’ gametocytes from the opposite sex. All the cultures were timed so that ‘exposed’ parasites were added to the cultures containing their ‘unexposed’ mates at the same developmental stage. For example, a final 5 ml culture could contain 15 µl of blood from a RNS exposed Pbs48/45ko infection (in which females are the ‘exposed’ sex) and 60 µl of blood from a Pbs47ko infection (in which ∼4 times more males are provided as ‘unexposed’ mates). We also set up cultures in vector mimicking media to verify that ‘unexposed’ parasites from each line are unable to produce ookinetes on their own. We recorded the densities of ookinetes as described for experiment 1.

### Statistical analysis

We used linear mixed effects models (R version 2.7.0; The R Foundation for Statistical Computing; www.R-project.org) because, by treating each infection (or pair of infections in Experiment 3) as a ‘random’ effect, we can account for problems associated with pseudoreplication arising from repeated measurements of each infection. In order to meet the assumptions made by parametric tests we arcsine square root transformed response variables where necessary. We minimised models following stepwise deletion of the least significant term and using log-likelihood ratio (*χ^2^*) tests to evaluate the change in model deviance until only significant terms remained, and we present F-ratios for fixed effects remaining in minimal models. We then re-ran minimal models using restricted maximum likelihood to estimate the effect sizes reported in the text. Unless otherwise indicated, data and estimated effect sizes are presented as proportions of the focal parasite stage produced in a given treatment, relative to that produced across all treatments for each infection.

### Theoretical model

We assume an infinite host population, divided into infected and uninfected individuals, with infected hosts containing a single infection producing haploid gametocytes that circulate in the blood. We assume that *q* gametocytes are transferred from host to vector during blood feeding, and that these gametocytes form a single mating group. The expected proportion of males in the mating group is *z*, i.e. the sex allocation strategy of the parasite strain that contributed the gametocytes. Hence, the actual number of males is a random variable *α∼Bi*(*q*,*z*) (i.e. binomially distributed with *q* trials and probability of success *z*). Consequently, the number of female gametocytes is *q*-α. Male and female gametocytes are killed with probability *d_M_* and *d_F_* respectively, leaving *Γ∼Bi*(*α*,*1-d_M_*) surviving males and *φ∼Bi*(*q-α*,*1-d_F_*) surviving females. We assume every surviving male produces *χ* gametes, and every surviving female produces a single gamete. We consider that male gametes are killed with probability *δ_M_*, and hence *γ∼Bi*(*χΓ*,*1-δ_M_*) male gametes enter the mating pool. We assume that all *φ* female gametes enter the mating pool (death of female gametes is formally equivalent to that of female gametocytes, and hence is implicitly included in the parameter *d_F_*). Therefore, the number of zygotes is equal to the number of gametes of the limiting sex, i.e. *ζ = min*(*γ*,*φ*). Finally, we assume that only a proportion *p* of zygotes are viable, due to either: (a) factors that kill each zygote with probability *Ω_Z_*; (b) factors acting on gametocytes resulting in the production of dysfunctional gametes at rate *Ω_M_* for males and *Ω_F_* for females; or (c) factors acting on gametes and causing them to become dysfunctional at rate ϖ_M_ for males and ϖ*_F_* for females, i.e. *p* = (*1-Ω_Z_*)(*1-Ω_M_*)(*1-Ω_F_*)(*1-ϖ_M_*)(*1-ϖ_F_*). In this context, we use the term ‘dysfunctional’ to refer to a gamete that achieves fertilisation but carries sufficient damage to render the resulting zygote inviable (i.e. unable to develop as an ookinete). Inviable zygotes will result when one or both of the parental gametes are dysfunctional. Hence, the number of viable zygotes produced by the mating group is *W* = *ζ p*, and this is our measure of fitness [15], [31], [32].

## Supporting Information

Figure S1 Evolutionarily stable sex allocation strategies when sex- and stage-specific mortality rates vary (χ = 1). Effect of male and female gametocyte mortality and male gamete mortality on the ES gametocyte sex ratio (z*), for a clonal population, when the number of gametes per male gametocyte (χ) is 1. On each plot, z* varies with male gamete mortality rate (δM). The coloured lines represent different gametocyte group sizes (q): 2 (grey), 5 (blue), 10 (red), 20 (green) and ∞ (yellow). Every plot depicts different parameter combinations of male gametocyte (dM = 0.1; 0.5; 0.9) and female mortality rate (dF = 0.1; 0.5; 0.9), with dM increasing left to right and dF increasing bottom to top.
(0.16 MB PDF)

Figure S2 Evolutionarily stable sex allocation strategies when sex- and stage-specific mortality rates vary (χ = 4). Effect of male and female gametocyte mortality and male gamete mortality on the ES gametocyte sex ratio (z*), for a clonal population, when the number of gametes per male gametocyte (χ) is 4. On each plot, z* varies with male gamete mortality rate (δM). The coloured lines represent different gametocyte group sizes (q): 2 (grey), 5 (blue), 10 (red), 20 (green) and ∞ (yellow). Every plot depicts different parameter combinations of male gametocyte (dM = 0.1; 0.5; 0.9) and female mortality rate (dF = 0.1; 0.5; 0.9), with dM increasing left to right and dF increasing bottom to top.
(0.21 MB PDF)

Figure S3 Evolutionarily stable sex allocation strategies when sex- and stage-specific mortality rates vary (χ = 8). Effect of male and female gametocyte mortality and male gamete mortality on the ES gametocyte sex ratio (z*), for a clonal population, when the number of gametes per male gametocyte (χ) is 8. On each plot, z* varies with male gamete mortality rate (δM). The coloured lines represent different gametocyte group sizes (q): 2 (grey), 5 (blue), 10 (red), 20 (green) and ∞(yellow). Every plot depicts different parameter combinations of male gametocyte (dM = 0.1; 0.5; 0.9) and female mortality rate (dF = 0.1; 0.5; 0.9), with dM increasing left to right and dF increasing bottom to top.
(0.20 MB PDF)
