# Supplementary material for: Sex and Death: The Effects of Innate Immune Factors on the Sexual Reproduction of Malaria Parasites
Source: PLoS Pathog. 2011 Mar 3;7(3):e1001309. doi: 10.1371/journal.ppat.1001309 (PMC3048364; doi:10.1371/journal.ppat.1001309)
Supplement: Figure S3 — Evolutionarily stable sex allocation strategies when sex- and stage-specific mortality rates vary (χ = 8). Effect of male and female gametocyte mortality and male gamete mortality on the ES gametocyte sex ratio (z*), for a clonal population, when the number of gametes per male gametocyte (χ) is 8. On each plot, z* varies with male gamete mortality rate (δM). The coloured lines represent different gametocyte group sizes (q): 2 (grey), 5 (blue), 10 (red), 20 (green) and ∞(yellow). Every plot depicts different parameter combinations of male gametocyte (dM = 0.1; 0.5; 0.9) and female mortality rate (dF = 0.1; 0.5; 0.9), with dM increasing left to right and dF increasing bottom to top. (0.20 MB PDF) [file ppat.1001309.s003.pdf]

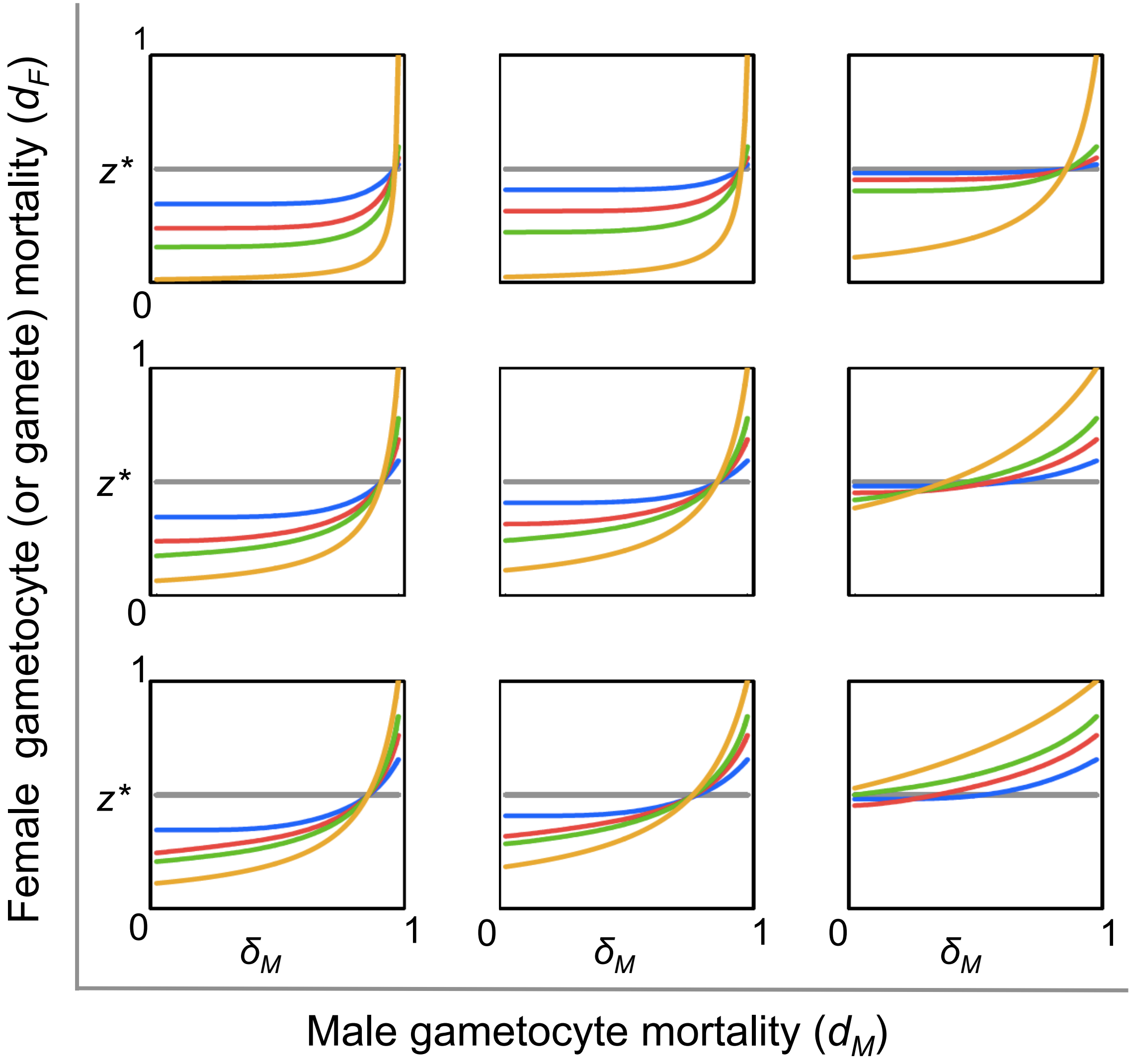

Figure S3. Evolutionarily stable sex allocation strategies when sex- and stage-specific mortality rates vary (χ=8). Effect of male and female gametocyte mortality and male gamete mortality on the ES gametocyte sex ratio (z^*), for a clonal population, when the number of gametes per male gametocyte (χ) is 8. On each plot, z^* varies with male gamete mortality rate (δ_M). The coloured lines represent different gametocyte group sizes (q): 2 (grey), 5 (blue), 10 (red), 20 (green) and ∞ (yellow). Every plot depicts different parameter combinations of male gametocyte (d_M = 0.1; 0.5; 0.9) and female mortality rate (d_F = 0.1; 0.5; 0.9), with d_M increasing left to right and d_F increasing bottom to top.
